# Solitary fibrous tumors of the pleura with Doege-Potter syndrome: a case report and three-decade review of the literature

**DOI:** 10.1186/1756-0500-7-515

**Published:** 2014-08-11

**Authors:** Wen Meng, Hong-Hong Zhu, Hu Li, Guoqing Wang, Dongshan Wei, Xing Feng

**Affiliations:** Department of Cardiothoracic Surgery, Hangzhou First People’s Hospital, Hangzhou, 310006 Zhejiang Province P R China; Department of Public Health, College of Health and Human Service, Western Kentucky University, Bowling Green, KY 42101 USA

**Keywords:** Solitary fibrous tumors of pleura, Doege-Potter syndrome, Surgery, Case report review

## Abstract

**Background:**

No case of solitary fibrous tumor of the pleura with Doege-Potter syndrome has been reported in China. This study was to report a rare repeatedly recurrent case of solitary fibrous tumor of the pleura with Doege-Potter syndrome diagnosed in China and a three-decade literature review of solitary fibrous tumor of the pleura with Doege-Potter syndrome worldwide.

**Case presentation:**

A rare case of solitary fibrous tumor of the pleura with Doege-Potter syndrome was diagnosed in 2005 with follow-up to 2011. All medical records were collected and literature of solitary fibrous tumor of the pleura with Doege-Potter syndrome from 1979 to 2011 was obtained through Medline. This typical case, diagnosed and confirmed by histopathologic results, was a 72-year-old Chinese woman who had a complaint of night sweat for a month. A localized mass 12 cm × 11 cm × 8 cm in size was found associated with pleural effusion in her left low chest cavity, and blood tests showed severe hypoglycemia. Removal of the mass solved the hypoglycemia. The case was repeatedly recurrent in April, 2010 and March, 2011 and had no signs of recurrence up to the end of 2011 after surgery. A review of 45 cases of solitary fibrous tumor of the pleura with Doege-Potter syndrome compared and summarized clinical characteristics, treatments, and outcomes by benign and malignant tumor nature.

**Conclusions:**

Incidence of solitary fibrous tumor of the pleura with Doege-Potter syndrome is similar between genders. There are no significant differences in clinical characteristics between benign and malignant cases. Surgery is the first effective treatment for solitary fibrous tumor of the pleura with Doege-Potter syndrome and the completeness of the initial resection is the key to preventing recurrence. Routine follow-up examinations are recommended for early detection of recurrence.

## Background

Solitary fibrous tumor of the pleura (SFTP) is a rare primary tumor, occupying less than 5% of all pleural tumors [1]. It arises from the submesothelial mesenchymal layer. Hypoglycemia accompanying SFTP is specifically referred to as the Doege-Potter syndrome (DPS) [2]. A large form of insulin-like growth factor 2 (IGF-2), which is probably an incompletely processed molecule of IGF-2, is derived from the tumor and considered to be a hypoglycemic mechanism [3]. England reported pathologic differences between benign and malignant SFTP among 141 benign and 82 malignant neoplasms [4] but no summary of clinical characteristics of benign and malignant SFTP with DPS due to a small number (n = 12) of DPS cases. Our report presented a typical case of repeatedly recurrent malignant SFTP with DPS in China, followed from 2005 to 2011. We then reviewed the literature of case reports of SFTP with DPS from 1979 to 2011 through Medline, and compared and summarized clinical characteristics, treatments, and outcomes by benign and malignant nature.

## Case presentation

A 72-year-old Chinese woman was hospitalized with a complaint of night sweat for one month in January, 2005. A localized mass 12 cm x 11 cm x 8 cm in size was found associated with pleural effusion in her left low chest cavity. Blood tests showed she was severely hypoglycemic. Removal of the mass solved the hypoglycemia. No other treatments were given. The histopathologic result showed a solitary fibrous tumor, potentially malignant.

The patient was followed up until April, 2010 when symptoms of night sweats returned. A physical examination showed she had no breath sound in the left low cavity, and the percussion note was dull on the left side of her thorax. Laboratory evaluation revealed no significant abnormalities in hematological or biochemical profile except low blood glucose. The complete resection of the mass revealed multiple well-capsulated masses in the left low chest cavity, the largest one 9 cm × 5 cm × 4 cm in size. Local tumor necrosis and mitosis up to 4/10 high power fields were microscopically identified. In March, 2011, the patient again had symptoms of night and morning sweats, and computed tomography and X-ray revealed a large recurrent tumor mass. The extensive excision of mass plus extensive excision of the pleura was performed revealing multiple recurrent parietal pleural masses in the left low chest cavity, the largest measured 14 cm × 10 cm × 10 cm. The episodes of hypoglycemia abated postoperatively and the patient seemed in good health after the surgery and had no signs of recurrence when we followed her up to the end of 2011.

### Literature review

We reviewed the literature in both English and Chinese languages from 1979 to 2011 through Medline and summarized 45 cases of SFTP with DPS and classified them into 22 benign and 23 malignant cases based on histopathologic results, referring to the criteria by England *et al.* [4].

### Summary of the 45 cases of SFTP with DPS

#### Basic characteristics

The age at diagnosis among 22 benign patients ranges from 38 to 79 years old, with a mean age of 60.8 ± 10.0 (standard deviation) and 23 malignant patients 31 to 79 years with a mean age of 64.1 ± 10.8. Twenty-four cases (12 benign and 12 malignant) are females and 21 (10 benign and 11 malignant) males. About 86.4% of benign and 87.0% of malignant SFTP patients are diagnosed at the ages of 50–70 years old (Table 1).

**Table 1:** **Summary characteristics of 45 cases of solitary fibrous tumors of the pleura with Doege-Potter syndrome, 1979-2011**

Characteristic	Benign (n = 22)	Malignant (n = 23)	***P***-value*
Age at diagnosis (years): Mean ± SD^$^	60.8 ± 10.0	64.1 ± 10.8	0.29
Gender			
Male	10	11	0.87
Female	12	12	
Sign			
Pleural effusion	5 (n = 16)	4 (n = 16)	0.69
Clubbing in fingers	5 (n = 16)	4 (n = 16)	0.69
Skin change	2 (n = 16)	1 (n = 16)	0.54
Tumor mass			
Clear borders	13 (n = 13)	16 (n = 20)	0.09
Location			
Right	13 (n = 21)	14 (n = 23)	0.94
Left	8 (n = 21)	9 (n = 23)	0.94
Up	1 (n = 17)	2 (n = 20)	0.65
Low	16 (n = 17)	18 (n = 20)	0.65
Size (cm)			
≤ 10	0 (n = 17)	1 (n = 21)	
>10	17 (n = 17)	20 (n = 21)	0.36
Morphologic Features			
Homogeneous	2 (n = 5)	3 (n = 17)	
Heterogeneous	3 (n = 5)	14 (n = 17)	0.29

*Pearson *P*-values for all frequency variables were estimated by individual Chi-square test and for age at diagnosis by *t*-test.

^$^SD: Standard deviation.

### Clinical characteristics

All patients have hypoglycemia-related symptoms. Abnormal consciousness, confusion, and sweating are often seen in both benign and malignant patients. Signs (such as pleural effusion, clubbing in fingers, and skin change) are also seen in both benign and malignant patients (Table 1).

From the 13 benign and 20 malignant reports that have a detailed description of the tumor mass, 100.0% benign and 80.0% malignant have sharply circumscribed borders. Most tumors are located in the right low area of chest cavity. The size of most benign and malignant tumors is greater than 10 cm. The average dimension of these SFTPs with DPS is 21.7 ± 5.0 cm for benign and 17.6 ± 4.1 cm for malignant tumors. There is no significant difference in morphologic features between benign and malignant tumors (Table 1).

### Treatments and outcomes

Of the 18 benign cases of SFTP with DPS with detailed surgical information, 13 (72.2%) are removed by simple excision of mass, and five cases by complex excision that included adhesion tissue. Of the 20 malignant cases with detailed surgical information, 11 (55.0%) are removed by simple excision, and 9 (45.0%) cases by complex excision. No distant and lymph node metastasis are found in all cases. The follow-up time in 29 (64.4%) of 45 cases ranges from 1 to 288 months (average time, 35.3 ± 57.2 months). All but four (two in benign and two in malignant) of the patients are free of recurrent SFTP with DPS. One recurrent tumor is performed by complex excision (recurrent tumor and adherent tissue). One recurrent tumor is performed by recurrent tumor excision. But they all are experienced repeated relapses. The other two patients refuse medical advice based on poor lung function and other reasons. Radiotherapy and chemotherapy are used in four malignant cases - two with single radiotherapy and chemotherapy but none of them show any effect or benefit. Two of three cases having detailed information of positron emission tomography-computed tomography examination show little to moderate uptake of [^14^C] fluorodeoxyglucose. Five of 20 cases given transthoracic needle biopsy could not get diagnosed, and seven of 20 cases could not be differentiated between benign and malignant SFTP due to limitation of tissue (Table 2).

**Table 2:** **Treatment and outcome of 45 cases of solitary fibrous tumors of pleura with Doege-Potter syndrome, 1979-2011**

Treatment/outcome	Benign (n = 22)	Malignant (n = 23)	***P***-value*
Treatment			
Surgery			
Simple excision	13 (n = 18)	11 (n = 20)	
Complex excision	5 (n = 18)	9 (n = 20)	0.27
Radiotherapy	0	1 (n = 23)	
Chemotherapy	0	1 (n = 23)	
Combination therapy			
Surgery + radiotherapy	0	2 (n = 23)	
Surgery + Chemotherapy	0	0	
Surgery + radio + Chemotherapy	0	0	
Recurrent	2 (n = 14)	2 (n = 15)	0.94
Imaging features			
Lobular borders	6 (n = 11)	9 (n = 15)	
Smooth borders	5 (n = 11)	6 (n = 15)	0.78
Pedicle	3 (n = 11)	5 (n = 16)	0.82
Calcification	2 (n = 9)	0	
Mass effect on mediastinum	8 (n = 9)	8 (n = 13)	0.16
Atelectasis	8 (n = 9)	7 (n = 9)	0.53
Chest wall involvement	1 (n = 12)	3 (n = 8)	0.11
Results of PET CT^$^	2 (n = 3)	0	
Benefit of transthoracic needle biopsy	8 (n = 11)	7 (n = 9)	0.80

*Pearson *P*-values for all frequency variables were estimated by individual Chi-square test.

^$^PET CT: positron emission tomography-computed tomography.

## Discussion

Some criteria, especially England [4], are used worldwide to distinguish benign from malignant SFTP [5]. The criteria defined by England *et al.* [4] include high cellularity, pleomorphism with cytonuclear atypia, more than four mitoses per ten high-power fields, associated necrotic or hemorrhagic areas. At least 80% of all SFTPs are benign, while the rest may show characteristics of malignant tumor [2, 4, 6]. From our study, there are roughly equal numbers of cases between benign and malignant SFTP with DPS which is higher than 12-13% of malignancy reported in literature [6]. Gender is nearly evenly distributed between the benign (12 women and 10 men) and malignant (12 women and 11 men), which is different from the results reported by England that DPS was three times as frequent in females as in males [4], probably due to the small number of DPS cases in his study. Most SFTPs associated with DPS are located in the right lower hemithorax, which is similar to England’s results [4]. The sizes of 100% benign and 95.2% malignant tumors are > 10 cm, similar to the conclusion from previous studies [2, 4].

Based on the information of 45 cases of SFTP with DPS in our study, the clinical performances between benign and malignant tumors are almost the same. Local recurrence can occur in both benign and malignant tumors of SFTP with DPS (two of 14 benign and two of 15 malignant). Surgery is the first effective treatment for both the original and recurrent benign and malignant masses [7–9]. These findings are also confirmed by our case report. Also, we think the completeness of initial resection is the key to preventing recurrence of SFTP with DPS. Some recurrent patients may lose the chance to get re-excision due to unfavorable health condition after the surgery. For the tumors located in parietal pleura, complete resection plus extensive excision of the pleura should be adopted in order to prevent recurrence. It is noteworthy that four cases (three malignant, one benign) performed transformation from non-hypoglycemia to hypoglycemia and one recurrent case performed malignant transformation 25 years after the surgery [10]. Routine follow-up examinations are recommended for early detection of recurrent SFTP. Our study also supports the finding that neither radiotherapy nor chemotherapy has any effect on the treatment of SFTP with DPS.

## Conclusions

Incidence of SFTP with DPS is similar between genders. There are no significant differences in clinical characteristics between benign and malignant cases. Surgery is the first effective treatment for SFTP with DPS and the completeness of the initial resection is the key to preventing recurrence. Routine follow-up examinations are recommended for early detection of recurrent SFTP with DPS.

## Consent

Written informed consent was obtained from the patient for publication of this Case Report and any accompanying images. A copy of the written consent is available for review by the Editor-in-Chief of this journal.

## Abbreviations

SFTP: Solitary fibrous tumors of pleura
DPS: Doege-Potter syndrome
IGF-2: Insulin-like growth factor 2.
